# Ambulatory chemotherapy: Past, present, and future

**DOI:** 10.1177/1078155220985916

**Published:** 2021-01-18

**Authors:** Racha Sabbagh Dit Hawasli, Stephen Barton, Shereen Nabhani-Gebara

**Affiliations:** Faculty of Engineering and Computing, School of Pharmacy and Chemistry, 4264 Kingston University, London, UK

**Keywords:** Ambulatory chemotherapy, elastomeric infusion pump, Covid-19, central access device, oncology

## Abstract

Ambulatory chemotherapy allows the delivery of short and extended chemotherapy infusions through a portable pump from the comfort of patients’ homes. It is essential to offer it for suitable candidates to ensure both their safety and the success of the treatment session. This requires a delicate balance between clinical assessment and patient acceptance.

The two main components of this treatment modality are the pump and the access device.

There are several pump designs and mechanisms on the market, with the latest being the portable disposable elastomeric one.

Clinicians along with a multidisciplinary medical team often decide upon the type of access device; patients are also involved whenever shared decision making is practiced.

Despite some reports of pump programming errors or malfunctions, research is underway to find innovative solutions to support its use.

## Background and definitions

Historically, the provision of chemotherapy infusions was solely delivered on an inpatient basis on oncology wards. For the past decades, the worldwide growing cancer incidence increased the strain on hospital resources as well as the patient travel and waiting times, thus affecting their treatment experience.

Breakthrough in oncology clinical practice gradually shifted chemotherapy provision to the outpatient settings and later closer to home, thus optimising hospital capacity and improving patient satisfaction. The choice of the setting and route of administration of the treatment depend on the prescribed chemotherapy protocol and medical status of the patient^1^ (Table 1).

**Table 1.** Innovations and developments in chemotherapy treatment.

Chemotherapy treatment	Definition
One-day hospital unit/Outpatient care	Outpatient hospital unit reserved for patients requiring short intravenous infusions over few hours on a daily, weekly or monthly basis^3^
Mobile chemotherapy units (MCUs)	These mobile units are driven to a specified location conveniently close to patients to deliver chemotherapy treatments beyond the second session. MCUs are managed by the same staff operating in the outpatient care. In 2007, the first MCU was launched in the UK^4^
Home chemotherapy/hospital-at-home care	Administration of intravenous chemotherapy at home under direct medical supervision by a specialist nurse; suited for patients wanting to avoid admission to the outpatient care or oncology ward for infusions lasting hours to days^3^
Oral chemotherapy drugs	Chemotherapy formulated as capsules and tablets currently available for many cancers^5^
Ambulatory chemotherapy	Delivery of chemotherapy outside the hospital using a portable infusion pump connected to patients at the hospital. This modality enables patients to receive continuous infusions lasting up to 7 days while freely ambulating^6-8^

These treatment patterns have had an erratic uptake between and within countries. Whereas treatment at the one-day chemotherapy unit is well established in most continents, ambulatory and home chemotherapy are more seen in developed and Western countries, such as Canada and the United Kingdom.^1,2^

Ambulatory chemotherapy (AC) allows the delivery of short (5-7 hours) and extended (1-5 days) chemotherapy infusions through a portable pump from the comfort of patients’ homes.

The connected infusion pump might need to be replaced during the course of treatment depending on its capacity and the length of the continuous infusion. This is carried out by a visiting home nurse, by the patients or carers at home, or during another hospital visit. There are different types of portable infusion pumps on the market with variable specifications. These could be connected to the patients either peripherally or through a central access device.

The purpose of this review is to present ambulatory chemotherapy delivery and share relevant practice experiences. This review will cover the two main components of ambulatory chemotherapy, the pump and the central access devices; the different types, their safety, and experience to date. The review will also shed light on the necessary assessment for patients deemed suitable candidates for AC, and the impact of the latter on healthcare delivery.

## Patient assessment for ambulatory chemotherapy

It is essential to offer AC for suitable candidates to ensure both their safety and the success of the treatment session. This requires a delicate balance between clinical assessment and patient acceptance.

Clinical assessment is comprised of five checkpoints, the first of which is a stable clinical status allowing safe hospital discharge. Performance scales such as the Eastern Cooperative Oncology Group (ECOG) performance scale or Karnofsky performance scale could be used for this purpose.

The second checkpoint relates to the suitability of the chemotherapy agent for ambulatory delivery. Published pilot studies on Ifosfamide, Trabectidin, and Fluorouracil confirmed that these could be safely administered on an ambulatory basis without the need for direct continuous medical monitoring.^3–5^ Newton et al. lists further chemotherapy regimens administered as case studies at University College London Hospitals National Health Service (NHS) Foundation Trust.^1^

The third checkpoint is having a means of communication such as a mobile phone to facilitate access to support in case of emergency.

Ability for self-care and management of side effects comprises the fourth checkpoint. It entails a clean home environment confirmed by the patient with cooperative family member(s) willing to accompany patients during the treatment sessions and learn support measures.

Proximity to a hospital is the fifth checkpoint ensuring access to support in case of emergency. A 30-60-minute travel time is acceptable, otherwise patients are invited to stay in a nearby hotel.

Patients’ acceptance for AC is portrayed in their consent to receive AC especially that they will be actively involved in the treatment. Patients’ choice in only implied in cultures and countries where shared decision making is practiced. A qualitative research conducted with cancer patients in Lebanon, a small Mediterranean country, assessed the diffusion of AC. Results showed that lack of patient empowerment led to oncologists taking all treatment related decisions on their behalf.^6^

Recently, telehealth platforms extended care management outside hospital wards. Remote monitoring of AC patients is undergoing extensive research in order to optimize patient experience and safety.^1,7–9^ This telemonitoring opportunity provides virtual support and a sense of security to patients as demonstrated in several studies.^10,11^

As such, when adequately assessed for AC, treated patients achieve improved health outcomes.

## Infusion pumps

Infusion pumps are mainly classified according to the below attributes:

- Operation mechanism; mechanical, electronic
- Pumping mechanism design; elastomeric, spring, vacuum
- Delivered product; chemotherapy, insulin, analgesics, etc.
- The environment where it is used; hospital/bedside, ambulatory
- Safety features; alarm, software

As such, the choice of pump lies in the detailed assessment of its use and context; a pump connected to a patient planned for discharge should most importantly be lightweight and free of programming errors, whereas a high risk medication should be delivered through a pump offering a high level of accuracy.^12^

Additional considerations include the patients’ ability to handle the connected pump, its availability at the hospital where the patient is receiving treatment, its cost, and insurance coverage.^13^

Portable pumps have been introduced to alleviate pressure from hospital premises and support ambulatory treatment.^7^ In the late 1970, a preliminary disposable portable infuser was used to deliver bleomycin and 5 Fluorouracil (5FU) in the ambulatory setting. Despite the technical complications, mainly related to inconsistencies in the flow rate, the absence of severe complications was a promising finding calling for technical enhancements to the infuser.^14^

Since then, advancements in pump technology made ambulatory chemotherapy more reliable and safer to use. Portable electronic and mechanical pumps were designed each offering different advantages.

There are two main types of portable infusion pumps; the battery operated and the disposable mechanical ones.

### Battery operated smart pumps

Also known as peristaltic pumps, these are programmable battery-operated infusion pumps with sizes of around 4 cm X 10 cm X 14 cm and weighing around 500 g. An attached medication cassette that holds the infusion bag is situated at the bottom of the screen and input pad.

In addition to being safe and simple to use, these pumps can provide assessment graphs and reports, and are equipped with alarms to notify errors such as low battery power, occlusion, faults, and air in line. These pumps also provide a high flow rate accuracy (±2.5-6%), and offer no restrictions on the volume and rate of infusion.^12,13,15–17^

The name “smart pump” was adapted owing to two main software components; dose error reduction systems (DERS) and drug libraries. Drug libraries are customized according to the practice at each hospital and ward to hold predefined parameters of the drug’s dose limits, strengths and other specifications. As such, the pump supports the user in setting up the correct rate and halts the infusion in case of errors in entering the data, miscalculations, or troubleshoots such as occlusion. Depending on the severity of the encounter these are referred to as soft or hard limits, with the latter requiring confirmation by a clinician to override the alert.^17,18^

A systematic review published in 2014 concluded that using smart pumps to infuse medications could decrease programming errors but not eliminate them. One of the retrieved studies found that pumps detected 79% of the encountered errors, and two other studies evaluating the severity of these errors classified them as “serious”, “catastrophic” or “potentially dangerous”.^18^

Ohashi et al. further describes the advantages of these pumps in supporting the nurses with dose calculations and adjustments, as well as connecting to the barcoding systems. For optimal use, this pump should be coupled to continuous quality improvement processes entailing the use, review and continuous update of the drug library.^18^

On the other hand, these pumps are associated with high risk of programming errors during set up, and patients often complain of the noisy alarm. Considering their weight and size, these are mostly used in-hospital as they have limited portability.^13^

In light of the current Covid-19 pandemic, the U.S. Food and Drug Administration (FDA) issued a report authorizing the use of infusion pumps and their accessories to treat Covid-19 infected patients. The FDA also required that manufacturers of pumps provide guidance on the cleaning of these reusable devices, most importantly, to recommend cleaning material readily available to patients for use at home.^19^ Consequently, thorough and adequate cleaning of any reusable pump should be considered and thus requiring additional cost and time.

Patients are advised not to shower/bath while a battery operated pump is connected; alternatively, a sponge bath is recommended.^20^

### Disposable mechanical pumps

These pumps use non-electric power to exert pressure on the fluid contained in the reservoir causing it to elute through narrow tubing into the patient’s body. There are three types of disposable mechanical pumps; positive pressure, negative pressure, and elastomeric (Table 2). Each type of pump has a certain driving force, but all are regulated by a flow restrictor which is a small part either taped to the patient’s skin or left to sense the room temperature. The pump is calibrated to infuse at a certain flow rate considering the temperature sensed by the flow restrictor.^21^

**Table 2.** Types of disposable mechanical pumps.

	Positive Pressure	Negative Pressure	Elastomeric
Mode of action	As the drug reservoir of the pump gets filled with the solution, it compresses the spring contained within, which in turn exerts pressure on the infusion bag driving the solution out through the tubing	The vacuum or area of very low pressure in one chamber of the pump becomes aggravated as the opposite chamber gets filled with the solution. The significant difference in pressure between the two chambers causes the movable wall plunger to push the fluid out through the tubing	A stretchable elastomer intended to hold the solution is contained within an outer protective shell made of hard plastic or a softer material. Once filled with the drug solution, the membranes of the elastomer exert pressure on the solution driving it out through the tubing
Disposable parts	Entirely disposable, or reusable	Entirely disposable	Entirely disposable
Accuracy	+/- 10 to +/- 20	+/- 10%	+/- 15%

Disposable pumps have many advantages including their light weight, small size, simplicity of use, independence from an external power supply, elimination of programming errors, disposability and low cost.

The rate of infusion and thus accuracy and duration of therapy are affected by temperature, atmospheric pressure, and fluid viscosity

Fluid viscosity, unlike temperature, is inversely proportional to flow rate.^22^ For that, these pumps are often calibrated with 0.9% sodium chloride or 5% dextrose; the two commonly used solvents to achieve the flow rate stated on the pump.

Other environmental conditions affect the performance of the pump, for example atmospheric pressure was found to be positively correlated with the flow rate.^23^

Partial filling of the pumps has been found to increase the flow rate, whereas storing elastomeric pumps at low temperatures hardened the elastomer thus decreasing the flow rate. It is therefore recommended to allow the refrigerated pumps to return to room temperature before connecting it to the patient.

The disadvantages of disposable pumps are thus mainly related to inconsistencies in flow rate and accuracy.^21^

Patients with a connected portable infusion pump are advised to keep the pump at room temperature, not to submerge it in water while showering, and to keep it at the level of the abdomen when sleeping. Alternatively, patients are advised to place the pump under the pillow during sleep, and to cover it with a plastic bag and hang it outside the tub while showering. Patients are also encouraged to frequently monitor the progress of the infusion and to report any delay or faster than expected emptying of the pump. It is advisable to provide patients with a leaflet on the precautions of use and emergency numbers to contact in case of anything unusual.^24–26^

## Safety of infusion pumps

Considering that hazardous solutions are infused through these pumps, malfunctions could have deleterious effects on patient safety. Despite the established safety of AC, several incidents of missed, over- or under-infusions have been reported due to user error or to deficiencies in the design, engineering, or components of the pump in turn leading to user error.

Between 2005 and 2009, the FDA received 56,000 reports of adverse events involving serious injuries and death resulting from mechanical, software, or programming errors related to the pumps.^27^ The most commonly reported incidents were due to defects in the software, user interface, mechanical or electrical functioning of the pump.

Reported incidents have been divided to six categories, 1) software problems, 2) alarm errors, 3) inadequate user interface design, 4) broken components, 5) battery failures, 6) electrical malfunctions.

Problems in the software could lead to either an inoperable pump in the absence of a recognisable cause, or a misinterpretation of the input. As such, a 10 mL/hour rate could be registered by the device as 100 mL/hour.

There is potential to encounter false positive or false negative alarms; for example, the device might fail to signal an occlusion in the tube or generate an alarm where there is no troubleshooting.

User interface refers to the way a computer and a user interact^28^ and when inadequate, such a design could lead to error. Discrepancies in the units, especially in the absence of system checks could lead to inadequate dosage or rate entry.

There are reports of erroneous flow rates, sparks, charring and shock when using a broken or damaged pump or from power failures, especially when poor maintenance is devoted to these devices.^29^

Consequently, the FDA published a white paper in 2010 entitled “Infusion pump Improvement Initiative” requesting enhancements on the level of the manufacturing process, the design of the device, and spreading awareness amongst users in order to promote safety.^30^

Notwithstanding the various safety alerts and vigilant risk assessment and validation processes fostered, incidents of malfunctions and toxicities were still encountered. These are mainly traced back to “human errors” in calculations and pump set up or choice of the pump.

In 2007, the Institute for Safe Medication Practices (ISMP) published a newsletter discussing the factors that led to the death of a 43-year-old Canadian patient who received high-dose fluorouracil via an ambulatory infusion pump. The programable pump, lacking a smart system to detect errors, was set to deliver the infusion over 4 hours instead of 4 days. Although the patient reported back to the clinic soon after the infusion had ended, no specific management of the overdose was undertaken which led to their death.

Multifactorial events had led to this; there was an error in the calculations of the first nurse and an inadequate double-checking process by the second nurse.

Additionally, the design of the pharmacy label was misleading as it states the rate in mL/24 hour first then in mL/hour.

Moreover, the nurses have a complex workload entailing several back to back high-risk tasks to be performed and the pump lacks an error reduction assistance.

Further investigations revealed that an inexperienced nurse had programmed the pump. Finally, the lack of insight on the adequate management of chemotherapy overdose led to irreversible harm brought upon the patient by the pancytopenia, hemodynamic collapse, and multiorgan failure.^31–33^

In another article for the ISMP, three accidental overdoses of fluorouracil in 2015 were described.

The first report describes how an error in programming a smart pump led to infusing a 2-day treatment over 2 hours. Despite the severe adverse events experienced, adequate management resulted in the patient’s complete recovery.

Another incident was reported whereby a 4-day infusion of fluorouracil was administered in less than an hour. This error was traced back to the technician confusing the yellow labels of the 2 ml/hour elastomeric infusion pump (EIP) and the 250 ml/hour pump. A different nurse noticed the empty pump and corrective measures were started with the antidote uridine triacetate.

The third report described a 5-fold fluorouracil overdose in a patient as a result of miscalculation by the pharmacist after the double-checking process was overlooked. The error was detected on the day of disconnecting the EIP and the patient was adequately managed.^31^

In 2010, the NHS published the “Design for patient safety: A guide to the design of electronic infusion devices” which addressed possible encounters and limitations with the programmable pumps and provided recommendations to manufactures and users. These recommendations aim at enhancing the design, functionality, and use of the pumps resulting in a better patient experience.^34^

In 2017, recommendations to prevent errors from the use of EIP induced by the staff or patients were delineated by both the ISMP and the South Australian Government.

First, the enforcement of clear prescribing information and maintenance of skilled healthcare professionals involved in the delivery of toxic therapeutic regimens. These should be adequately informed of the components of the pump, the connection and disconnection process, storage conditions, and patient education.

Second, it is highly recommended to use pumps that reduce the chances of errors through alerts and flow rate limits thus assisting in the verification of the correct infusion details.

Third, using a single type of ambulatory pump within the organisation and providing education on the connection process further reduces the rates of error and keeps the staff familiar with the device.

Fourth, checklists and double verification by a healthcare practitioner, patient or family member are advised in preventing errors.

Fifth, careful choice of patients for AC able to manage the pump in line with the provided instructions, and educating them about their treatment, dose, and duration of the infusion as well as the reporting of unusual side effects should be practiced.

Finally, clear and standardized labels on the final preparation from the pharmacy department must be adopted.^31^

## Central access device

Appropriate vascular access and device selection for cancer patients should be assessed by the treating oncologist. The literature lacks strict guidance in this perspective as a variety of factors define the most appropriate access route including amongst others; patient status (age, comorbidities), the frequency and duration of treatment, the type of chemotherapy prescribed, patient preference and engagement in self-care, cost, and reimbursement schemes. The practice, therefore, varies between using peripherally and centrally inserted catheters.^35–37^

A recent survey conducted with Canadian oncologists and oncology nurses specialized in breast cancer assessed their access practices as well as their perceptions of risks and complications. Results showed that physicians are the main decision makers in this perspective and take into consideration prior IV access and feedback from the nursing team. The observed trend was that vesicant drugs were preferably infused via a central access device (CAD), however, nurses recommended the use of CAD in all cases as they believed it is correlated with enhanced patient quality of life and conserved their veins. Despite the recognized complications related to both access routes and the lack of best practice guidance, CAD remains the preferred access route for oncologists.^38^

### Rational for using central access devices

In addition to saving patients the anxiety and pain from repetitive venipuncture and cannulation, central access devices serve as a long-term access to oncology patients requiring frequent and prolonged infusions. As these patients often require treatment over months to years, repetitive peripheral access, routinely replaced every 3-4 days, might reduce vein patency over the treatment duration.^39^

Moreover, vesicant and irritant chemotherapy drugs have the potential to cause injection site reaction and resulting serious extravasation. They require rapid dilution when injected which is achieved by infusion through central veins.^39,40^ On the other hand, the infusion flow and rate necessary to avoid extravasation increases the fragility of small peripheral veins with a low threshold for this complication.^41^

Prevention of extravasation trough use of CAD has been warranted; for example, it is always recommended to infuse Trabectidin through a CAD.^42,43^

Published guidelines from the European Society of Medical Oncology (ESMO) and the American Society of Clinical Oncology (ASCO), recommend a Central Venous Access for oncology patients;

“*Long-term central venous access devices are essential in the management of oncology patients, as they minimise the discomfort of frequent venepuncture and cannulation*”.^36^

“*The management of the patient with cancer demands stable venous access that is used for a wide range of indications*”.^44^

In what concerns AC, and since its main objective is for cancer patients to regain normalcy, patients connecting the pump will be actively ambulating and their movements, including those of their hands, unrestricted. In this perspective, CAD is more fit for use than peripherally inserted catheters to ensure habitual activity and avoid dislodgement. As such, clinical practice of renowned cancer centres recommend CAD for home infusion.^45–48^

The use of CAD also allows patients connecting a portable infusion pump to disconnect it at home thus avoiding a return trip to the hospital. For example, the Dana-Farber Cancer Institute and the Johns Hopkins Kimmel Cancer Center provide detailed online instructions to support patients in this task.^49,50^

This is essential at current times of Covid-19 pandemic when renowned institutions have issued guidelines and statements to decrease its impact on patients’ cancer care continuum. Home treatment coupled to telehealth have been put forward as an acceptable solution to safeguard patient safety while providing timely treatment sessions.^51^

### Complications of central access devices

The use of CAD is associated with immediate and late complications, both of which should be prevented and well managed by a multidisciplinary specialized team. Preferably, this team would be comprised of medical oncologists/hematologists, registered nurses, interventional radiologists, surgeons and infectious disease specialists.^36,52^ Pharmacists are also an important pillar of this team as they are strategically positioned to offer support to patients. Community pharmacists are within reach to the public at the front end of healthcare services, whereas clinical pharmacists on hospital wards are proactively involved in discharge counselling to mitigate medication error.^53^ Pharmacists can provide counselling on pump handling, care for CAD, and uncovers the truth behind treatment related myths and misconceptions. Additionally, pharmacists can signpost patients to Non-Governmental Organisations (NGO’s) for support and social reinforcement.

The insertion technique is responsible for the immediate complications during catheter placement which can rapidly escalate to critical conditions (Table 3). Ultrasound guidance has shown to drastically reduce the incidence of immediate complications.^36,44,52^

**Table 3.** Immediate and delayed complications of CAD.

**Immediate Complications**	
Vascular	Arterial injury
	Arterial puncture
	Venous injury
	Bleeding
	Hematoma
Cardiac	Arrhythmia
	Cardiac arrest
Pulmonary	Pneumothorax
	Pneumomediastinum
	Chylothorax
	Tracheal injury
	Injury to the recurrent laryngeal nerve
	Air embolus
**Delayed Complications**	
Device dysfunction	Fibrin sheath formation
	Fracture
	Thrombosis
	Central venous stenosis
Infection	Sepsis
	Shock
	Death

On the other hand, it could take weeks to years for the onset of delayed complications; related morbidity and mortality is greatly reduced with early recognition (Table 3). Device dysfunction is often treated with fibrinolytics or catheter removal.^44,52^

CVC related infections have a high incidence in cancer patients and are classified as either local (at insertion site) or systemic (blood stream infection), thus called catheter related blood stream infections (CRBSI). Upon clinical manifestations of an infection, cultures are taken, and antibiotic therapy is started. CVC removal is indicated when infections result in sepsis, endocarditis, and port abscess, as well as when patients do not respond to 48-72 hours of antibiotic. Cultures positive for Staphylococcus aureus, fungi or mycobacteria also warrant CVC removal. Studies reported lower infections rates with port-a-caths compared to tunnelled catheters and peripherally inserted central catheters (PICC) lines.^36^ Coagulase-negative staphylococci, Staphylococcus aureus and Candida spp are the main cause of CRBSI.

### Types of central access devices

There are three mains types of CADs classified according to the way they are connected to the vessel (tunnelled, non-tunnelled, implanted), their insertion point (subclavian, jugular, etc.), and finally their characteristics (infused with antibiotic, antiseptics or other solutions) (Table 4).

**Table 4.** Types, description and features of the three main types of central access devices.

Types of CAD	Description	Features
Tunnelled Catheter	Catheter inserted by tunnelling under the skin into the subclavian or internal jugular vein. The part used to administer or withdraw fluids remains outside the body (e.g. Hickman, Broviac)	Serves for a long period and is adequate for administration of fluids such as chemotherapy, blood products and parenteral feeding.No needle sticks required
Peripherally Inserted Central Catheter (PICC)	Line inserted into a large vein in the arm (hence the name peripheral) and advanced forward into the subclavian vein	Could serve up to 12 months, no needle sticks required, placed at bedsideAssociated with a higher risk of thrombosis which explains their short sustainability
Implantable port (Port-a-cath)	Port placed completely below the skin consisting of a chamber component, raised disk of around 1-inch diameter underneath the skin, and a connected thin flexible tube extending into a major vein. Access is ensured using a special needle, Huber needle, inserted into the chamber on one end, and connected to the syringe or infusion on the other end	Long term patency while maintaining low infection risks

### Care for central access devices

Whenever the CAD is not in use, usually between treatment sessions, an antibiotic lock therapy is recommended to prevent infection of the biofilm. A highly concentrated antibiotic is combined with an anticoagulant and injected into the catheter to remain indwelling until it is accessed.^36^

Infections, thrombosis, and occlusion are possible complications of the CAD, however, the current treatment guidelines offer detailed regimens for the prevention and treatment of these events.^36,37,44,54^

In their recently updated guidelines, the Centers for Disease Control (CDC) listed 20 items for the prevention of intravascular catheter-related infections and maintenance.^54^

It is essential to designate trained healthcare personnel for managing patients with CAD as well as perform periodic assessment of knowledge.

The guidelines also recommend careful selection of catheter type and insertion site based on the intended purpose, treatment duration, and patient medical history.

Aseptic technique and adequate protective equipment were also described for performing catheter insertion and care. This also extends for skin preparation, catheter site dressing regimen as described with chlorhexidine and sterile gauze.

The guidelines clearly advise to “*Use a chlorhexidine/silver sulfadiazine or minocycline/rifampin -impregnated CVC in patients whose catheter is expected to remain in place >5 days*”.

Systemic antibiotic prophylaxis is, however, not recommended before or during CAD insertion. Specific topical antibiotics and antiseptics are recommended for patients undergoing haemodialysis only.

The CDC advises against the routine replacement of CVC and PICC lines, even in the presence of fever. The replacement of administration sets should be done every 96 hours to 7 days.^54^

Patients should be advised not to submerge the catheter in water and showering should only be permitted if the catheter and its entry point is protected with an impermeable cover. Patients are also counselled to report any discomfort or unusual observation, and to use a 2% chlorhexidine solution for daily skin wash.^54^ Patients should also be educated on infection signs so to report them the earliest.

## Impact of ambulatory chemotherapy

A robust national healthcare infrastructure is essential to foster a safe and well-established practice of AC. A multidisciplinary team of healthcare professionals is key to ensure a seamless experience for patients opting to receive AC. This is essential especially that AC cannot and is not planned to replace other treatment modalities.

However, at times of the current Covid-19 pandemic, healthcare is shifting delivery of treatments to the home setting. This is especially valuable for cancer patients considering their immunosuppression, and for hospital resources as it optimizes use of hospital beds. A recent publication of the ASCO Post describes a successful implementation of a chemotherapy home delivery program; careful assessment is done for suitable patients, protocols, and home environments.^55^ As such, AC is a practical alternative to support in delivering chemotherapy agents with long enough stability and requiring minimal support at home, such as 5FU.

AC has the potential to positively impact the patient’s quality of life and alleviate pressure from the carers and the treating hospital. AC has been renowned for giving back the patients a sense of normality and control over their symptoms and treatment.^56–58^

In 2002, a prospective pilot single centre study in the UK assessed the feasibility of administering the deGramont regimen on an ambulatory basis using EIP compared to inpatient treatment. The study also aimed at capturing patient acceptability for this treatment modality, and its effect on their quality of life.^59^

The deGramont regimen, often delayed for logistic reasons, involves administering a 2-hour folinic acid, a 30-minute bolus 5FU infusion, and a 22-hour 5FU infusion intermittently over 2 days. Twenty-six patients in total were recruited equally distributed between the outpatient and inpatient treatment groups.

The outpatient treatment delivered a 48-hour 5FU infusion through a CAD for suitable patients, while the inpatient treatment delivered it through a peripheral line at the hospital. Patients received adequate counselling on both treatment modalities and were given the choice of treatment.

The inpatients perceived many benefits for their treatment modality; feeling secure at the hospital especially in the lack of family support at home, avoiding long travel distances from home, fear from having a CAD. This treatment group complained from admission delays, long waiting times to receive treatment, and extension of hospital stay.

The outpatient treatment group appreciated the reduced commuting times, improved quality of life, sense of independence, being home, and satisfactory nursing care at home. Three out of thirteen patients developed CAD related complications but were successfully treated and all patients were satisfied with their choice of treatment and expressed adhering to it for future sessions.

The quality of life was measured using a validated tool; the European Organization for Research and Treatment of Cancer Quality of Life Core Questionnaire (QLQ-C30). Outpatient treatment was associated with better quality of life and less cost compared to inpatient treatment. In light of these benefits, outpatient treatment became standard of care at the centre.^59^

A retrospective single centre study in Korea evaluated the patient satisfaction and cost of ambulatory versus inpatient chemotherapy treatment with FOLFOX in 80 patients having similar baseline characteristics.

All patients were admitted to the hospital at least for their first cycle and then randomized to either ambulatory or inpatient treatment. The same medications were given in both treatments but with a slight variance in dosing. Inpatients received two 5FU infusions over 22 hours while ambulatory patients received one pump with the adequate dose of 5FU to infuse over 2 days.

Ambulatory treated patients expressed higher satisfaction than inpatients in terms of cost, level of discomfort, burden on family members, waiting time, worries about chemotherapy and understanding its schedule, and awareness of the potential side effects (p < 0.05). Although inpatients had to worry less about their injection site and experienced a better management of nausea episodes, overall satisfaction was still higher in the outpatient group. Additionally, AC incurred significantly lower costs compared to inpatient treatment.^3^

In a comparative study conducted in China between inpatient (N = 52) and ambulatory (N = 50) treatment, three factors were found to influence patient’s choice of treatment; educational level, family responsibilities, and employment. Patients in the ambulatory treatment group were more educated, more likely to be employed and be the primary caregiver of the family. These findings comply with the primary advantage of ambulatory treatment that is patients not being restricted to their hospital beds and carrying out their normal daily activities while receiving treatment. It was interesting to note that although patients were happier to receive ambulatory therapy, they did experience more fatigue, nausea and vomiting than their counterparts. The authors highlighted the need for appropriate medical counselling on lifestyle modifications and use of rescue oral therapy for chemotherapy induced nausea and vomiting.^7^

AC using portable infusion pumps is gaining momentum in Hong Kong as three recent publications have described its impact on patients’ quality of life and decreases pressure on healthcare resources.^57,58,60^ This was following a successful delivery of 29 infusions to 24 cancer patients between November 2017 and March 2019.

## Practice-based challenges with ambulatory chemotherapy and research guided solutions

The paucity of literature assessing the performance of EIP in clinical practice, encouraged researchers from Kingston University to conduct a two-phase audit between 2013 and 2014. The study aimed at evaluating the accuracy of EIP and patient satisfaction at three gastrointestinal units of the Royal Marsden hospitals through an observational study and a survey.

The observational study collected data required to assess the infusion duration, remaining volume (if any) at disconnection time, action taken in case of incomplete pump emptying at the pre-set disconnection time. Additional variables that could possibly affect pump accuracy were also collected such as type of chemotherapy infused, and the access device used.

The survey assessed patients’ experience and satisfaction with the pump.

5FU was mainly infused through CAD to patients aged 29-88 years.

Forty percent of calculated infusion rates were slower than those labelled, and 57% of these pumps were discarded with considerable remnant volumes of 5FU. These findings impact not only treatment outcomes but also nurses’ workload and patient waiting times. Researchers were, however, unable to collect percentages of pumps infusing faster than labelled as patients came before their scheduled visit.

Despite the high patient satisfaction scored, it is essential to mitigate the impact of pump accuracy on treatment outcomes.^61^

Consequently, a subgroup of these researchers collaborated with computer scientists from Kingston University to develop a software that uses digital images for assessing pump performance. By monitoring the remaining volume in the pump, the software generates an estimate of the end of infusion time. Preliminary results were promising and could support detecting inconsistencies in treatment delivery early enough to avoid serious complications.^62^

Another group of Canadian researchers and clinicians assessed the feasibility of weighing the 5FU pumps as an alternative to visual inspection when checking for residual volumes at disconnection (hour 46). Filled and empty pumps were weighed using a pharmacy and a kitchen scale. Weighing proved to be an acceptable estimate of the volume and time that have elapsed and are remaining for 5FU delivery even with a kitchen scale, readily accessible for patients. In addition to generating figures useful for these calculations, the study also proved that in many instances the remaining volume was greater than 10%, the renowned acceptable margin. This further highlights the importance of a practical tool for patients to monitor the rate of treatment delivery.^63^

The researchers built on these findings to conduct another study whereby patients were asked to present to the hospital midway through their treatment to check for the rate of infusion based on its weight. On average, the observed pumps had a flow rate (13.7%) greater than that labelled and its acceptable variability. However, estimated flow rates at each hour between 17 and 27 were lower than expected. This correlates with the declaration of the pump’s manufacturer that flow rates are not constant. This study reinforced the use of weight for monitoring drug delivery but not for estimating the end time of infusion due to the inherent variability of the flow rate.^64^

## Conclusion

Ambulatory chemotherapy is a feasible alternative to hospital stay based on patient and physician experience. Despite some reports of unfortunate incidents and malfunctions, AC has been the focus of recent research using novel ways to mitigate them. At current times of Covid-19 pandemic, it is essential to ensure patient care continuum while maintaining safety and positive experience. With careful considerations and an adequate infrastructure in place, AC has the potential to cater for these needs.

## Key points

- Ambulatory chemotherapy uses portable infusion pumps to deliver chemotherapy at home.
- Positive experiences using ambulatory chemotherapy warrants its use at times of Covid-19 pandemic.
- Recent research using technology and tools accessible for patient is addressing practice-based challenges of ambulatory chemotherapy.
